# *KRAS* mutations detected by the amplification refractory mutation system–Scorpion assays strongly correlate with therapeutic effect of cetuximab

**DOI:** 10.1038/bjc.2011.247

**Published:** 2011-07-05

**Authors:** H Bando, T Yoshino, K Tsuchihara, N Ogasawara, N Fuse, T Kojima, M Tahara, M Kojima, K Kaneko, T Doi, A Ochiai, H Esumi, A Ohtsu

**Affiliations:** 1 Department of Gastroenterology and Gastrointestinal Oncology, National Cancer Center Hospital East, 6-5-1 Kashiwanoha, Kashiwa, Chiba 277-8577, Japan; 2 Cancer Physiology Project, Research Center for Innovative Oncology, National Cancer Center Hospital East, 6-5-1 Kashiwanoha, Kashiwa, Chiba 277-8577, Japan; 3 Pathology Division, Research Center for Innovative Oncology, National Cancer Center Hospital East, 6-5-1 Kashiwanoha, Kashiwa, Chiba 277-8577, Japan

**Keywords:** ARMS/S, colorectal cancer, direct sequencing, formalin-fixed paraffin-embedded specimen, *KRAS*

## Abstract

**Background:**

We aimed to compare the sensitive and quality-controlled *KRAS* testing with direct sequencing and to assess the impact on decision making of treatment.

**Patients and methods:**

We analysed genomic DNA isolated from macrodissected formalin-fixed paraffin-embedded specimens by direct sequencing and an amplification refractory mutation system–Scorpion assay (ARMS/S) method. Cetuximab was administered to patients identified as having wild-type (WT) *KRAS* using direct sequencing. Therapeutic effects were evaluated according to their *KRAS* status as determined by ARMS/S.

**Results:**

Among the 159 patients, the overall mutation rate was determined to be 37.0% by direct sequencing and 44.0% by ARMS/S. For the patients diagnosed as WT by direct sequencing and treated with cetuximab (*n*=47), a response rate of 16.0% was observed for 38 ARMS/S WT patients, whereas 9 ARMS/S mutant (MUT) patients failed to respond. The ARMS/S WT patients showed significant improvement in progression-free survival (PFS) and overall survival (OS) compared with ARMS/S MUT patients (PFS median 5.0 *vs* 1.7 months, hazards ratio (HR)=0.29, *P*=0.001; OS median 12.1 *vs* 3.8 months, HR=0.26, *P*=0.001).

**Conclusion:**

Sensitive and quality-controlled *KRAS* testing may provide improved predictive power to determine the efficacy of anti-epidermal growth factor antibodies.

Retrospective subset analyses and prospective randomised phase III clinical trials have suggested that anti-epidermal growth factor antibodies do not benefit patients with metastatic colorectal cancer harbouring *KRAS* mutations (Amado *et al*, 2008; Karapetis *et al*, 2008; Tol *et al*, 2009; Van Cutsem *et al*, 2009). On the basis of these findings, regulatory authorities in Europe, the United States and Japan require pretreatment *KRAS* mutation testing. In Europe, the KRAS European Quality Assurance Program (http://kras.eqascheme.org/) has been launched and several Communauté Européene-labelled *KRAS* mutation test kits such as the TheraScreen K-RAS Mutation Kit (DxS-QIAGEN, Manchester, UK), KRAS LightMix (TIB MolBiol, Berlin, Germany) and PyroMark Q24 KRAS Kit (QIAGEN, Duesseldorf, Germany) have been approved for diagnostic use. The TheraScreen Kit combines the amplification refractory mutation system (ARMS) with a unique bifunctional fluorescent primer/probe molecule (Scorpion) and is recommended for clinical use because of its high sensitivity, robustness and convenience (Franklin *et al*, 2009; Jimeno *et al*, 2009; Kotoula *et al*, 2009; Whitehall *et al*, 2009; Angulo *et al*, 2010; Ogasawara *et al*, 2011).

Together with these standardised methods, direct sequencing is still one of the most accessible methods. However, several critical disadvantages of direct sequencing for diagnostic use have been indicated. These include its low sensitivity and lack of strict criteria for distinguishing mutant signals from contaminated noises. Furthermore, we have recently reported that insufficient PCR amplification further limits the sensitivity and specificity of direct sequencing. This is particularly important when DNA isolated from formalin-fixed paraffin-embedded (FFPE) specimens, which contain low levels of amplifiable DNA, is used (Bando *et al*, 2011). To increase the sensitivity of direct sequencing, macroscopic isolation of tissues in which cancer cells occupy >70% of the area (macrodissection) is recommended for preparation of genomic DNA (Kotoula *et al*, 2009).

Although discrepancies in interpretation between the ARMS–Scorpion assay (ARMS/S) and direct sequencing have been noted, the impact of these discrepancies on treatment has not been adequately evaluated (Franklin *et al*, 2009; Kotoula *et al*, 2009). In this study, we evaluated the validity of ARMS/S and direct sequencing by comparing the therapeutic effects of cetuximab in patients in whom *KRAS* mutations were analysed by these two methods.

## Patients and methods

### DNA samples and *KRAS* mutation testing

Genomic DNA was extracted from primary and metastatic colorectal cancer tissues of patients scheduled to receive cetuximab. DNA extraction from FFPE tissue blocks has been previously described. The *KRAS* exon-2 fragment was amplified and sequenced according to previously described methods (Bando *et al*, 2011). The *KRAS* PCR Kit (DxS-QIAGEN) was used for detection of seven major mutations in *KRAS* codons 12 and 13. Reactions were performed using the LightCycler 480 Real-Time PCR System (Roche Diagnostics, Mannheim, Germany) and analysed with LightCycler Adapt software v1.1 (Roche Diagnostics) as previously described (Bando *et al*, 2011).

### Patients

Cetuximab was administered at the National Cancer Center Hospital East (NCCHE) in patients diagnosed with wild-type (WT) *KRAS* by direct sequencing. Furthermore, *KRAS* mutation status was evaluated using ARMS/S.

Patients who met all inclusion criteria were retrospectively included in analyses. Inclusion criteria were as follows: (1) age ⩾20 years; (2) histologically confirmed adenocarcinoma of the colon or rectum; (3) presence of unresectable metastatic disease; (4) baseline computed tomography (CT) scan performed within the previous 28 days; (5) initial evaluation by CT scan within 3 months; (6) documentation of refractory to previous fluoropyrimidine, oxaliplatin and irinotecan administration; (7) *KRAS* mutational status determined by direct sequencing and ARMS/S; (8) Eastern Cooperative Oncology Group performance status score ⩽2; (9) adequate haematological, hepatic, renal and bone marrow function; and (10) undergone treatment with cetuximab monotherapy regimen or combination regimen with cetuximab plus irinotecan. In the monotherapy regimen, cetuximab was administered at an initial dose of 400 mg m^–2^, followed by weekly infusions of 250 mg m^–2^. In the combination regimen, cetuximab was administered at the same dose as for monotherapy, followed by biweekly infusions of 150 mg m^–2^ irinotecan.

The study was conducted with the approval of the institutional review board.

### Measured outcomes

The therapeutic response rate was evaluated according to the Response Evaluation Criteria in Solid Tumours (version 1.0). Progression-free survival (PFS) was defined as the time from the first cetuximab administration to either first objective evidence of disease progression or death from any cause. Overall survival (OS) was defined as the time from the first administration of cetuximab to death from any cause.

### Statistical analysis

The response rate, PFS and OS of all patients were revalued for this study. Fisher's exact test and the Mann–Whitney test were used to compare the patient characteristics and response rates. The PFS and OS data were plotted as Kaplan–Meier curves and the differences between the groups categorised by ARMS/S-identified *KRAS* status were compared by the log-rank test. The hazard ratio (HR) was calculated from the Cox regression model with a single covariate. All analyses were performed using IBM SPSS Statistics 18 package software (SPSS Inc., Tokyo, Japan).

## Results

### Mutation rates determined by direct sequencing and ARMS/S

From April 2009 to March 2010, 159 specimens were tested using both ARMS/S and direct sequencing (98 specimens were collected from NCCHE and 61 from other hospitals). Both methods had a success rate of 100%. In all, 59 (37.0%) *KRAS* mutations were detected by direct sequencing and 70 (44.0%) by ARMS/S (Table 1a). All mutations identified by direct sequencing were also identified by ARMS/S. However, 11 (7.0%) of the 70 *KRAS* mutations identified by ARMS/S were not detected by direct sequencing. The overall concordance rate of the two methods was 93.0% (Table 1b).

### Patient characteristics

From April 2009 to March 2010, 47 patients met with all of the inclusion criteria (11 patients were treated with cetuximab monotherapy and 36 patients were treated with cetuximab plus irinotecan). Of the 47 patients, 38 and 9 patients were identified by ARMS/S as WT (ARMS/S WT) and mutant (ARMS/S MUT), respectively (Table 2). Patient characteristics of the two groups (ARMS/S WT *vs* ARMS/S MUT) were not significantly different except for the incidence of lung metastases (Table 2).

### Response to treatment

The response rate of ARMS/S WT patients was 16.0%. In contrast, no objective tumour response was observed in ARMS/S MUT patients. In addition, the disease control rates (including partial response and stable disease) of ARMS/S WT and ARMS/S MUT patients were 66.0% and 56.0%, respectively (Table 3).

### Survival

**Figure 1:**
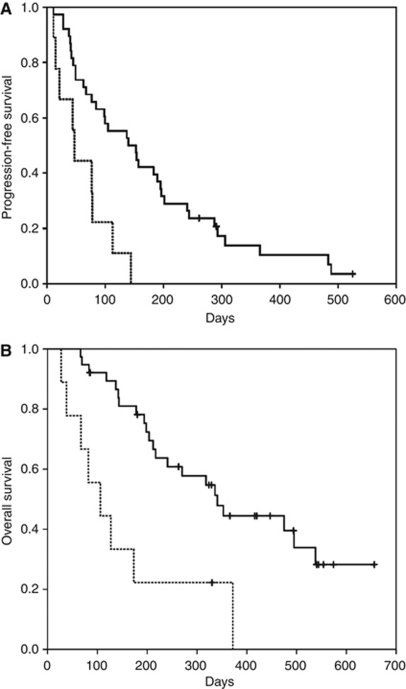
(**A**) Kaplan–Meier plots of progression-free survival (PFS) according to *KRAS* status determined by the amplification refractory mutation system–Scorpion assay (ARMS/S). For the patients treated with cetuximab-containing regimens, the median PFS values were 5.0 and 1.7 months for ARMS/S wild-type (solid line) and ARMS/S mutant (dashed line) patients, respectively. The difference was statistically significant (HR=0.29, *P*=0.001). (**B**) Kaplan–Meier plots of overall survival (OS) according to *KRAS* status determined by ARMS/S. For the patients treated with cetuximab-containing regimens, the median OS values for ARMS/S wild-type (solid line) and ARMS/S mutant (dashed line) patients were 12.1 and 3.8 months, respectively. The difference was statistically significant (HR=0.26, *P*=0.001).

The median PFS of the 38 ARMS/S WT and 9 ARMS/S MUT patients was 5.0 and 1.7 months, respectively (HR=0.29, *P*=0.001; Table 3 and Figure 1A). The relative dose intensity of cetuximab therapy was not significantly different between ARMS/S WT and ARMS/S MUT patients (Table 3). The median OS of the 38 ARMS/S WT and 9 ARMS/S MUT patients was 12.1 and 3.8 months, respectively (HR=0.26, *P*=0.001; Table 3 and Figure 1B).

When the patients were divided as per treatment regimen, the median PFS and OS of the patients treated with cetuximab plus irinotecan were significantly different from ARMS/S WT and ARMS/S MUT patients (PFS; HR=0.23, *P*=0.002, OS; HR=0.187, *P*=0.001). Similar trends were also observed for the patients treated with cetuximab monotherapy (PFS; HR=0.497, *P*=0.332, OS; HR=0.674, *P*=0.586).

## Discussion

The present guidelines for *KRAS* testing allow direct sequencing for MUT detection (Allegra *et al*, 2009). To overcome the low sensitivity of direct sequencing, we performed macrodissection of the tissues in order to enrich the tumour cell-derived DNA. We also improved the PCR conditions based on our previous study (Bando *et al*, 2011). The mutation rates determined by direct sequencing in the present study were comparable with those reported in previous studies (36.0–43.0%) as per various mutation detection methods, including ARMS/S, and thus support the validity of our direct sequencing procedure (Amado *et al*, 2008; Karapetis *et al*, 2008; Tol *et al*, 2009; Van Cutsem *et al*, 2009). In contrast, the mutation rate determined using simultaneous ARMS/S appeared to be higher than that found in previous clinical trials (Amado *et al*, 2008; Karapetis *et al*, 2008; Tol *et al*, 2009; Van Cutsem *et al*, 2009). Therefore, we surmise that enrichment of tumour cell-derived DNA may further enhance the sensitivity of ARMS/S.

Next, we examined whether this higher sensitivity could result in improved clinical relevance. The median PFS, OS and response rates of *KRAS* WT patients determined by ARMS/S were comparable with those previously reported (median PFS, 3.8 months; median OS, 9.5 months; response rate, 13.0%) (Karapetis *et al*, 2008). In contrast, the median PFS, OS and response rates of the *KRAS* MUT patients, although determined as WT by direct sequencing, were comparable with those of MUT *KRAS* patients reported in previous clinical trials (median PFS, 1.8 months; median OS, 4.5 months; response rate, 1.0%) (Karapetis *et al*, 2008).

Two factors may be responsible for the significant advantage of ARMS/S. First, the higher sensitivity of the ARM/S assay can detect the presence of a lesser number of *KRAS* mutations than direct sequencing. Second, strictly controlled criteria for MUT identification provided robust detection and eliminated the ‘grey zone’ cases that we often encountered using direct sequencing.

On the other hand, the intratumoral heterogeneity of tumour tissues for *KRAS* gene status suggested that residual *KRAS* WT tumour cells may respond to cetuximab, but this idea is still under debate (Baldus *et al*, 2010). In the present study, although ARMS/S MUT patients showed poorer PFS and OS than the WT patients, 5 of the 9 patients achieved disease stability in the first CT evaluation. Although this study had limitations such as small sample size and retrospective design that could have caused substantial biases, it appears that tumour heterogeneity allowed a reasonable level of disease control. Thus, further evaluation with an adequate sample size, in a prospective manner, would be required to determine which of the testing methods (direct sequencing or ARMS/S) would be a better predictive marker for clinical benefits.

In conclusion, our study suggested that *KRAS* mutation status determined by ARMS/S appeared to be more closely related to clinical effects than that determined by direct sequencing, although there were limitations of sample size and retrospective design. Whether *KRAS* mutation status determined by ARMS/S can be used as a predictive biomarker is not yet known. However, the study results warrant further investigation of this method, which should evaluate the correlations between *KRAS* mutation status and clinical outcomes in comparison with those achieved by direct sequencing.

## Figures and Tables

**Table 1a:** Comparison of mutation detection techniques

**Method**	**Direct sequencing**	**ARMS/S**
Success rate	100% (159 out of 159)	100% (159 out of 159)
Mutation rate	37.0% (59 out of 159)	44.0% (70 out of 159)

Abbreviation: ARMS/S=amplification refractory mutation system–Scorpion assay.

**Table 1b:** Pairwise comparisons of mutation detection frequency

	**Direct sequencing**		
**ARMS/S**	**WT**	**MUT**	**Total**
WT	89 (56.0%)	0 (0%)	89 (56.0%)
MUT	11 (7.0%)	59 (37.0%)	70 (44.0%)
Total	100 (63.0%)	59 (37.0%)	159 (100%)

Abbreviations: ARMS/S=amplification refractory mutation system-Scorpion assay; MUT=mutant; WT=wild type.

**Table 2:** Patient characteristics

	**DS WT**		
**Characteristic**	**ARMS/S WT (*n*=38)**	**ARMS/S MUT (*n*=9)**	***P*-value**
Treatment (cetuximab monotherapy/ cetuximab+irinotecan)	8/30	3/6	0.350^a^
Age (median)	65	66	0.234^b^
Sex (M/F)	26/12	6/3	0.604^a^
ECOG PS (0/1or 2)	29/9	4/5	0.740^a^
Site of primary cancer (right/left/rectum)	17/10/11	1/3/4	0.401^a^
Histologic appearance (well diff./poorly diff.)	34/4	9/0	0.414^a^
			
*Metastatic site*			
Liver (%)	47.0	44.0	0.586^a^
Lung (%)	47.0	89.0	0.026^a^
Nodes (%)	47.0	78.0	0.100^a^
Ascites (%)	21.0	11.0	0.433^a^
No. of metastatic sites (1/>2)	19/19	2/7	0.128^a^

Abbreviations: ARMS/S=amplification refractory mutation system–Scorpion assay; DS=direct sequencing; ECOG PS**=**Eastern Cooperative Oncology Group Performance Status Scale; F=female; M=male; MUT=mutant; WT=wild type.

a One-tailed Fisher's exact test.

b Mann-Whitney test.

**Table 3:** Efficacy and relative dose intensity in the test population according to *KRAS* status determined by ARMS/S

	**DS WT**	
**Characteristic**	**ARMS/S WT (*n*=38)**	**ARMS/S MUT (*n*=9)**
Partial response	6	0
Stable disease	19	5
Progressive disease	13	4
Response rate	16.0%^a^	0%^a^
Disease control rate	66.0%	56.0%
Progression-free survival, median (months)	5.0	1.7
Overall survival, median (months)	12.1	3.8
Relative dose intensity		
Cetuximab, median (range)	0.94 (0.57–1.00)	0.93 (0.57–1.00)

Abbreviations: ARMS/S=amplification refractory mutation system–Scorpion assay; DS=direct sequencing; MUT=mutant; WT=wild type.

a *P*=0.257 (one-tailed Fisher's exact test).
